# Design and Evaluation of Liposomal Sulforaphane-Loaded Polyvinyl Alcohol/Polyethylene Glycol (PVA/PEG) Hydrogels as a Novel Drug Delivery System for Wound Healing

**DOI:** 10.3390/gels9090748

**Published:** 2023-09-14

**Authors:** Hamide Hemati, Fateme Haghiralsadat, Mahdie Hemati, Ghasem Sargazi, Nastaran Razi

**Affiliations:** 1 Department of Biology, Faculty of Sciences, Yazd University, Yazd P.O. Box 81195741, Iran; hamidehemati64@yahoo.com; 2 Medical Nanotechnology and Tissue Engineering Research Center, Yazd Reproductive Sciences Institute, Shahid Sadoughi University of Medical Sciences, Yazd P.O. Box 89195999, Iran; 3 Department of Advanced Medical Sciences and Technologies, School of Paramedicine, Shahid Sadoughi University of Medical Sciences, Yazd P.O. Box 8916188635, Iran; 4 Department of Clinical Biochemistry, Faculty of Medicine, Shahid Sadoughi University of Medical Sciences, Yazd P.O. Box 8916188635, Iran; 5 Noncommunicable Diseases Research Center, Bam University of Medical Sciences, Bam P.O. Box 7661713669, Iran; g.sargazi@gmail.com; 6 Department of Biology, Faculty of Science, Science and Research Branch, Islamic Azad University, Tehran P.O. Box 1477893855, Iran

**Keywords:** hydrogel, nanoliposome, polyvinyl alcohol polymer, sulforaphane, wound healing

## Abstract

Hydrogel scaffold has been widely applied as drug delivery systems for treating skin injuries. However, the poor drug loading and rapid drug release of hydrogel restricted their application. In the current study, we present a nanoliposome containing sulforaphane (SF) as a nano-drug delivery system that is encapsulated within the scaffold hydrogel system to overcome these limitations and improve wound healing. The hydrogel substrate consisting of 10% polyvinyl alcohol (PVA)/5% polyethylene glycol 400 (PEG400) was prepared by the freeze–thaw method, and the nanoliposomal system was manufactured by the thin film hydration method at different molar ratios of cholesterol: SPC: DPPC: DSPE-PEG2000. The nanoliposome and hydrogel system was characterized by physicochemical analyses. The findings achieved from the optimization of the sulforaphane-loaded nanoliposome (SFNL) displayed an increase in the molar ratio of SPC, leading to a higher entrapment efficiency and a gradual release profile. Narrow size distribution, optimal electrical charge, and the lack of molecular interactions between SF and nanoliposome components in the FTIR analysis make SFNL a suitable drug delivery system for the wound healing process. The obtained SFNL-encapsulated freeze–thawed hydrogel system has sufficient and specific swelling ability at different pH values and increased mechanical strength and elongation. Additionally, the release pattern of SFNL at different pH values showed that the release of SF from liposomes depends on the pH value of the environment and accelerates in line with decreasing pH values. Encapsulation of nanoliposomal SF in the hydrogel structure provides a sustained release pattern of SF compared to its free form and increased as the pH environments continued to raise. The cytotoxicity and cell uptake of SFNL-loaded hydrogels against human skin fibroblasts (HFF cell line) were investigated. The in vitro analyses displayed that the toxicity properties of SF and SFNL were dose-dependent, and SFNL exhibited lower toxicity compared to free SF. Furthermore, the proper cell compatibility of the prepared hydrogel against the HFF cell line was confirmed by the MTT assay. These findings imply that the hydrogel scaffold loaded with SFNL may have wound-healing potential.

## 1. Introduction

Wound healing and its management depend on the extent of infection and inflammation, the capacity of blood vessel formation and cell proliferation, as well as the presence of chronic and acute ulcers. These elements can have an impact on four phases of wound healing: hemostasis, inflammation, proliferation, and remodeling [1]. A wound dressing is a substance that helps stop bleeding and wound debridement, promoting cell and vessel proliferation, while reducing the risk of infection. The most common types of wound dressings include cloth, foam, hydrogel, transparent, and hydrocolloid. Hydrogel scaffolds produced by natural or synthetic polymers are a hydrophilic polymer with a three-dimensional, cross-linked, and water-swollen network [2]. Hydrogels reacting to environmental changes, such as temperature, pH, ionic strength, and the presence of enzymes, are very important in drug delivery applications. They are able to hold a large amount of water without deterioration of their structure. So, they can be designed and functionalized to mimic biological tissues [3]. Hydrogels are non-toxic and do not cause immune or inflammatory responses. These mentioned properties make them suitable as tissue mimetic hydrogels and also as drug delivery systems to the target tissue [4,5]. They can stay in situ for up to 3 days and the research findings indicated that synthetic hydrogels were non-cytotoxic on normal cells [6,7,8]. Hydrogel has adhesive properties and adheres to the skin like tape. Yet, it can be covered by a bandage of gauze and depending on the extent of the wound, it was changed daily for heavily exuding wounds and 2–3 days for light ones. After the desired time, the edges of the hydrogel are lifted and gently removed from the wound. Polyvinyl alcohol (PVA) hydrogel, a synthetic polymer, indicates beneficial properties, including softness, transparency, free-toxicity, hydrophilicity, and biocompatibility [9]. Moreover, polymers such as polyvinyl alcohol are demonstrated to be biodegradable, with non-toxicity and low cost to treat injuries with the least side-effects [10]. Furthermore, PEG 400, possessing a plasticizing nature, was applied to boost the flexibility, swelling capacity, and thermal resistance of the PVA hydrogel structure [11]. Thus, the present study aimed to develop a novel PVA/PEG400 hydrogel that can encapsulate drugs and be used as an appropriate wound dressing. Complementary and alternative medicines such as using herbal extracts play a significant role in the treatment of wound and in reducing their complications. Sulforaphane (SF) is a natural isothiocyanate generated from cruciferous vegetables and has anti-microbial, anti-inflammatory, antioxidant, anti-diabetic, and anti-cancer properties. SF protects cells from oxidative damage, thereby the overexpression of nuclear factor-erythroid factor 2-related factor 2 (Nrf2), inducing antioxidant enzymes and lowering the levels of reactive oxygen species (ROS) [12,13]. The process of wound healing is significantly aided by SF, which acts as a ROS inhibitor and cell protector with antioxidant characteristics. SF ameliorates skin aging, ultraviolet-induced skin damage, and maintenance of collagen levels during photo-aging via the activation of the Kelch-like ECH-associated protein 1 (Keap1)-Nrf2 pathway, the inhibition of the activator protein-1 (AP-1), and the expression of metalloproteinases [14,15]. However, the therapeutic potential of SF is confined due to its linear and hydrophobic structure, poor solubility in water, and low bioavailability. Drug delivery systems with controlled and burst-free release features have been designed and developed to alter usage constraints [16,17]. A liposome is a spherical vesicle that encapsulates both hydrophobic and hydrophilic drugs. Similar cell membrane structure with non-toxicity, prevention of the premature inactivation of the drugs, accumulation of drug delivery to tissue, reduction in toxicity of encapsulated agents to normal tissues, carry a high concentration of the drug, ability to encapsulate both positively and negatively charged agents, the slow and contentious release kinetics of the drug at the target cells, and avoidance of the rapid phagocytosis of drugs are the main advantages of nanoliposomes [18,19]. Hence, the bio-distribution, pharmacokinetic index, and contentious release pattern of SF are improved in liposomal forms. Slow permeation of SF-loaded nanoliposome results in an effective and optimal dose of SF with few side effects on the wound area. In the current study, for the first time, we used the therapeutic potential of SF as herbal medicine in wound therapy. In order to overcome its limitations, we entrapped it in a nanoliposome (SFNL) and performed optimization to obtain the best nanoliposome characteristics. Then, the SFNL-loaded PVA/PEG400 hydrogel was designed and developed by incorporating SF nanoliposome into the hydrogel structures. This PVA/PEG400 gel-forming hydrogel containing nanoliposomal SF as a novel wound dressing formulation was manufactured to improve cutaneous wound healing. In fact, SF encapsulation in this new hybrid drug delivery system provides controlled and sustained release kinetics of SF at the wound site. This ability not only prevents the deactivation and premature failure of SF but also ensures the continuous release of SF from the double barrier of nanoliposomes and then the hydrogel structure. These features promote the release of SF for a long time, which decreases the frequency and high concentration of drug administration and local or systemic side effects.

## 2. Results and Discussion

### 2.1. Characterizations of SFNL and Hydrogel Scaffold

#### 2.1.1. Selection of Optimum Formula 

The development and commercialization of a wound dressing that effectively restores moisture to the injured area and prevents the spread of infection and stimulates the body’s own regeneration processes is a major goal. Therefore, the aim of the present study is to achieve an optimal formulation based on a 3D scaffold containing nanoliposomal SF to maximize the effectiveness and penetration of drugs. Liposomal SF was designed and prepared by the thin-film hydration method at different molar ratios of Chol: SPC80: DPPC to overcome poor water solubility and hydrophobicity of SF. As shown in Table 1, the %EE of SF is decreased when the cholesterol content is increased since cholesterol incorporation reduces drug entrapment by competing with the drug to enter the lipid bilayer, thus preventing lipophilic drugs from entering the vesicles [20,21]. Phospholipids with long alkyl chains increase the percentage of drug entrapment in the following order, F3 > F2 > F1 (Table 1). Increased drug encapsulation and reduced drug release can be achieved by increasing the molar ratio of phospholipids with more alkyl chain lipids and increasing the hydrophobic area. This effect can be attributed to the position of sulforaphane as a hydrophobic drug in the lipid bilayer membrane, which increases the interaction of drugs with the lipid chain of phospholipids. Our finding was consistent with previous studies conducted in this field [22,23]. In order to prepare the long-circulating and stealth liposomes, DSPE-PEG2000 (5%) was added to the formulations [24]. Studies showed that DSPE-PEG2000 could improve the skin penetration of SF-loaded nanoliposomes by increasing the hydrophilicity, flexibility, and stability of nanoliposomes and promoting the wound healing process [25,26]. As displayed in Table 1, F3 was selected as an optimal formulation to be used for further experiments. 

#### 2.1.2. Particle Size, Biodistribution, and Zeta Potential of Optimized Formulation

**Figure 1:**
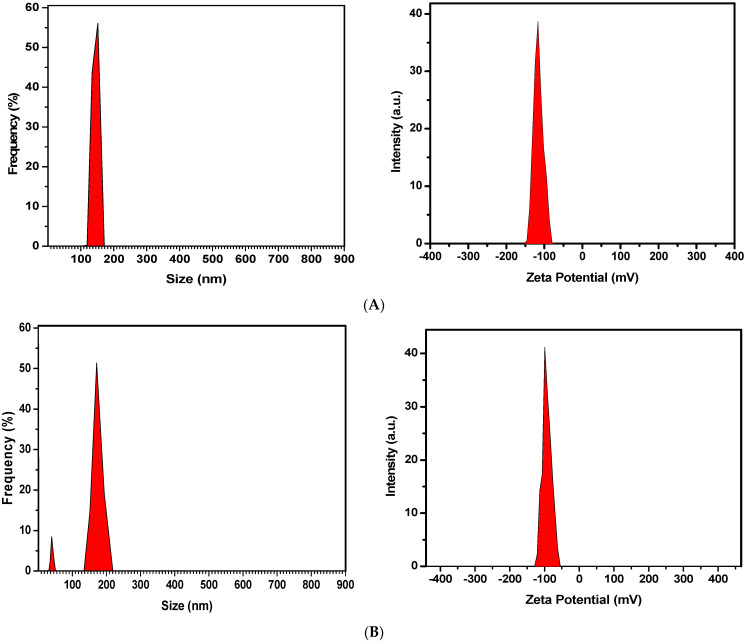
The particle size and zeta potential of the optimal formulation; (**A**) blank nanoliposome; (**B**) SF-loaded nanoliposomes.

The size of the blank nanoliposome revealed an average diameter of 113.8 ± 2.7 nm with a high negative charge potential of −110.6 ± 4.5 mV. An increase in size and a slight decrease in surface charge were observed in SF-loaded nanoliposomes, which were 125.0 ± 2.3 nm and −94.6 ± 0.21 mV, respectively (Figure 1). A zeta potential over +30mV or −30 mV is needed to prevent nanovesicle aggregation and increase stability. These electrostatic repulsive forces between particles can prevent them from sticking together and provide information about the dispersion index and vesicle size of the nanoliposomes [27]. On the other hand, the net negative charge of the formulations provided a high affinity for SF during encapsulation. Ibaraki et al. evaluated the effects of the zeta potential of liposomes on cellular uptake and intradermal penetration. They manufactured various liposomes based on cationic, neutral, and anionic surface charges and demonstrated that the dermal drug delivery of liposomes is increased with negative surface charge compared to cationic and neutral formulations [28,29]. As a novel topical drug delivery technology, hydrogel containing anionic liposomes is utilized to enhance skin penetration and transport across the stratum corneum [30,31]. Therefore, SFNL with negative zeta potential is expected to improve intracellular absorption and intradermal permeation of SF.

#### 2.1.3. The Release Profile of SFNL and Hydrogel Containing SFNL

**Figure 2:**
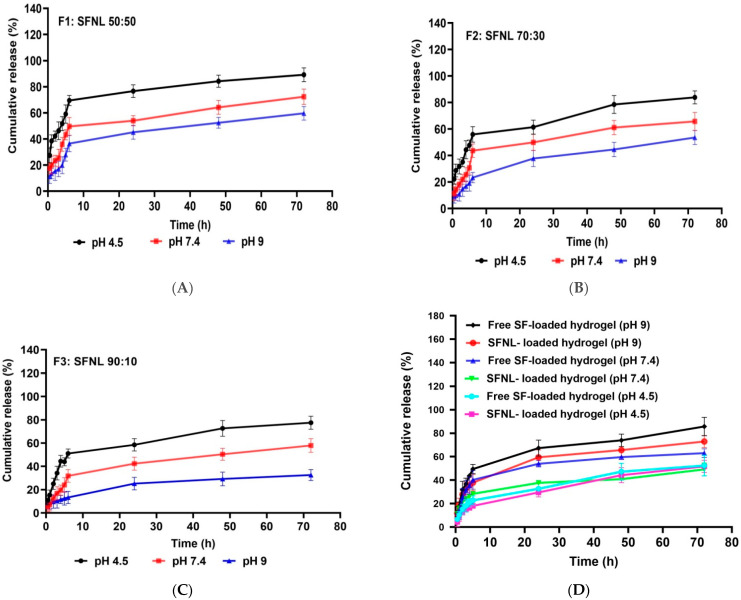
The drug release profile; SFNL at different molar ratios of phospholipids: cholesterol: 50:50 (**A**), 70:30 (**B**), and 90:10 (**C**) at various pH values (4.5, 7.4, and 9); and the release pattern of free SF and SFNL-loaded hydrogel at pH 4.5, 7.4 and pH 9 (**D**) at 37 °C.

Safe and efficient delivery with an appropriate therapeutic concentration of the drug without overdosing and side effects depends on the release rate of the drug from the wound dressing to the wound milieu. Various factors, especially the wound pH, display an important factor in the drug release amount and the healing process. The pH of the wound shifted to an acidosis environment during the healing of acute wounds and alkaline milieu during the healing of, chronically infected wounds. Such changes in wound pH can influence the dissolution of the drug, the structure of nanoliposome and hydrogel scaffold, and their effect on the drug release pattern from a delivery system. Thus, for preeminent wound management, we selected different pH values to simulate this situation in the wound healing process. In this study, the release amount of SF-loaded nanoliposomes at various pH values (4.5, 7.4, and 9) was evaluated. The in vitro analysis was simulated in the physiological condition (pH 7.4), acidic microenvironment (pH 4.5), and alkaline pH like in the chronic wound microenvironment (pH 9) at 37 °C. As shown in Figure 2, SFNL has the highest release rate at a pH value of 4.5 compared with other pH values at all-time points. In fact, the acidic conditions led to an elevated SF release compared to neutral or alkaline environments. In the process of wound healing, pH changes from neutral to acidic [32]. Therefore, when the wound environment becomes more acidic (pH < 6), it causes changes in the electrical charge and structure of the nanoliposome components, leading to better fusion of nanoliposomes to the cell membrane and faster drug release and intracellular delivery [33,34]. Uppal et al. showed that in acidic pH conditions, the solubility of the drug increases, and a higher release rate takes place [35]. Studies have shown that the pH of chronic wounds shifts toward an alkaline pH with a slower healing rate [36]. Our findings showed that the release rate of SF decreased in an alkaline pH medium, followed by a slower release profile. As shown in Figure 2C, liposomal formulations containing 10% cholesterol exhibited the lowest extent of drug release, 77.50, 57.88, and 32.61 h after incubation at pH 4.5, 7.4, and 9, compared to formulations containing 50% cholesterol (Figure 2A), which released 89.17, 72.36, and 59.69 h after incubation at pH 4.5, 7.4, and 9, respectively. This phenomenon states that an increase in phospholipid content as the ratio of phospholipid to cholesterol increases with fatty acid chain length decreases the rate of drug release, whereas cholesterol increases the level of drug release by interfering with the regular structure of the liposome membrane [37]. In all formulations, SF was released with an initial moderate burst in the first 6 h followed by slower release rates up to 72 h. This pattern was crucial to maintain the level of SF continuously at the wound site to increase the efficacy and therapeutic index of SF. It is crucial to investigate the biocompatibility of PVA/PEG 400 hydrogel for the transport of nanoliposomal SF to the wound site and to compare its loaded and unloaded release properties at different pH values (4.5, 7.4, and 9). It was observed that the release of SF from the hydrogel increased with increasing pH values (Figure 2D). At 72 h, the hydrogel with free SF loaded at pH 4.5, the cumulative drug release rate was only 52.34%, while it reached 63.08% and 85.79% at pH 7.4 and pH 9, respectively. The SFNL released from the hydrogel scaffold was also investigated and the cumulative drug release rates at pH 4.5, 7.4, and 9 were reported to be 51.72, 49.20, and 72.95%, respectively. These findings were consistent with the swelling behavior of free SF and SFNL-loaded hydrogel in PBS solution at different pH values. Because of the ionization of the functional groups at higher pH and the electrostatic repulsion between them, the swelling rate of the hydrogel system accelerates, resulting in a faster increase in drug release rate in neutral and alkaline conditions than in acidic milieu [38]. According to the findings, the amount of release from the SFNL-loaded hydrogel was much less than that of the free SF-loaded hydrogel. Joraholmen et al. incorporated resveratrol into a liposomes-in-hydrogel delivery system. Their results demonstrated a continuous and sustained resveratrol release from liposomes. In addition, consistent with our findings, the incorporation of liposomes into the hydrogel matrix had a synergistic impact on the release rate of resveratrol [39]. In all formulations, SF was released with an initial moderate burst in the first 6 h followed by slower release rates up to 72 h. This pattern was crucial to maintain the level of SF continuously at the wound site to increase the efficacy and therapeutic index of SF. Therefore, the SFNL-loaded hydrogel had pH-sensitive and slow-release behavior, which makes it a suitable wound dressing.

#### 2.1.4. Fourier-Transform Infrared Spectroscopy (FTIR)

**Figure 3:**
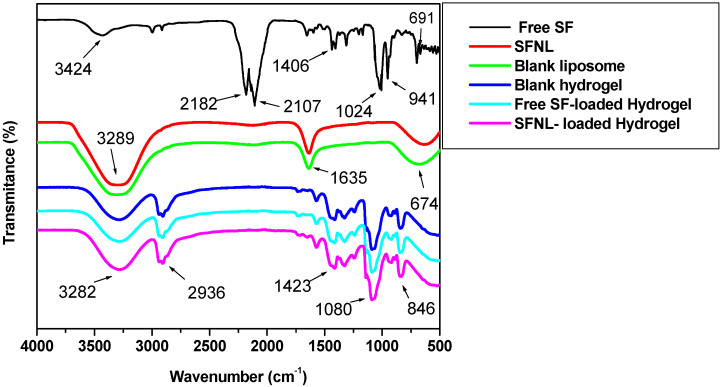
FTIR spectra of free SF and optimized formulations; nanoliposomes and PVA/PEG400 hydrogel with/without drugs.

FTIR spectra were used to prove that SF was loaded by a lipid bilayer and a blank hydrogel and also that there were no molecular interactions between SF, nanoliposome, and hydrogel components. The FTIR spectra of liposomes loaded or unloaded with SF, blank hydrogel, SF-laden hydrogel, and SFNL-loaded hydrogel are shown in Figure 3. The presence of peaks at 3240–3356 cm^−1^ is related to the stretching vibrations of alkyl groups, while the peaks at 1646–1628 cm^−1^ are assigned to the stretching vibrations of alkyl ester groups in nanoliposomes. The FTIR spectrum of both free SF and SFNL-loaded hydrogel was obtained as well as the blank hydrogel. There were characteristic peaks for free SF at 2182 and 2107 cm^−1^ that exhibited the existence of the N=C=S group. The peaks at 1406 cm^−1^, 1024 cm^−1^, and 691 cm^−1^ verified the stretching vibration of C−S, C−N, and S=O bonds in free SF, respectively. The patterns of FTIR spectra of blank PVA/PEG400 demonstrate a broad peak at 3324–3255cm^−1^, indicating the O-H stretching vibration. Peaks can also be observed in FTIR spectra of PVA at 2936 cm^−1^, 1642 cm^−1^, and 1080 cm^−1^, which are related to C–H, C=O, and C-O stretching vibrations, respectively. C–H bending vibration is indicated in the region of 1420–1443 cm^−1^. In fact, the hydroxyl group demonstrated in the region of 3324–3255cm^−1^ indicates water retention and swelling capacity in the polymeric hydrogel, while C–H stretching and bending denote hydrocarbon chromophores in PVA/PEG400 hydrogel. These FTIR spectra of all formulations showed that there was no new molecular interaction and cross-linking between PVA/PEG400 and SF compared to the unloaded and loaded hydrogel. 

#### 2.1.5. X-ray Diffraction (XRD)

**Figure 4:**
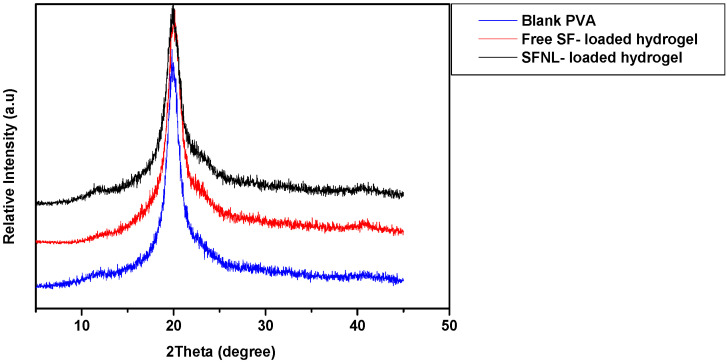
The XRD patterns of PVA/PEG400 hydrogels synthesized under optimal conditions.

XRD analysis was used to determine the crystal information of PVA/PEG 400 hydrogel, a free hydrogel with SF, and hydrogel loaded with SFNL. Blank hydrogel represents the characteristic diffraction intensity at an angle of 19.8° and a less intense shoulder at an angle of 40.9° (Figure 4). This diffraction pattern shows the semi-crystalline structure of PVA [40,41]. Blank PVA/PEG 400 has a comparable diffraction peak with the same intensity for both free hydrogels with SF and hydrogel loaded with SFNL. This event demonstrates that not only was there no undesired molecular interaction between free SF/SFNL and PVA/PEG 400 hydrogel, but they were also well dispersed and homogenous in the PVA matrix environment. Moreover, the free SF and SFNL do not interfere with their crystalline properties. This finding was also in line with previous studies [42,43]. 

#### 2.1.6. Determination of Swelling Ratio (%)

##### Swelling Ratio of Hydrogel Scaffolds

**Figure 5:**
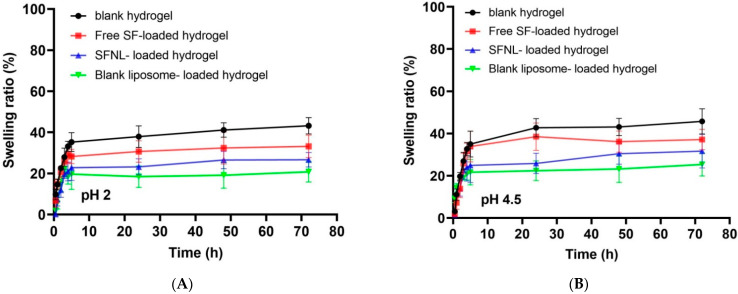

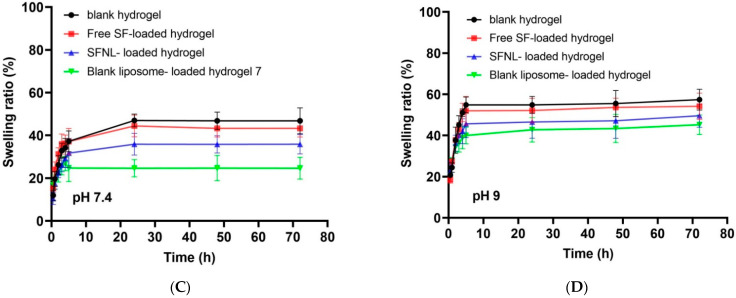
The swelling ratio of freeze-dried PVA/PEG400 hydrogels in phosphate-buffered saline (PBS); (**A**) pH 2, (**B**) pH 4.5, (**C**) pH 7.4, and (**D**) pH 9.

As shown in Figure 5, it is clear that the swelling percentage of PVA hydrogels after 72 h was more than 45%. As expected, the swelling ratio displayed a negative correlation with the encapsulation rate. At pH 7.4, the swelling ratios of the free SF and SFNL-loaded hydrogels reached the lowest at 43.2 and 35.8, respectively (Figure 5C). According to the results, all samples reached their maximum swelling ratios after 5 h. The polymer hydrogel without drugs had the greatest swelling ratio after 24 h, followed by polymer films loaded with free SF since it is a hydrophobic molecule and its addition to polymer films caused less water absorption and a lower swelling ratio. Additionally, the addition of SF-containing nanoliposomes and blank nanoliposome to polymer films resulted in a lower swelling ratio. Bavarsad et al. prepared and characterized the griseofulvin-loaded liposomes in the form of chitosan films. They exhibited that the addition of liposomes containing griseofulvin as a hydrophobic compound to polymer films led to lower water absorption and swelling ratio [44]. Entezam et al. investigated the physicomechanical properties of PVA/PEG/clay nanocomposite hydrogels containing curcumin. They discovered that hydrogels’ low swelling ratio might be related to their hydrophobic drugs and the interaction between nanoparticles and polymer chains, which makes the gel network denser and inhibits the polymer layer from aspirating water molecules [45]. The percent swelling ratio of blank liposome-loaded hydrogel was lower than that of free SF and SFNL-loaded hydrogel (Figure 5). This is due to the increase in cross-linking density and thus the decrease in pore size, which is inversely related to the water absorption of blank liposome-containing hydrogels [46]. In the free form, the drug initially swelled more than the blank hydrogel. Due to the absorption of more water, the hydrogel containing free SF is gradually deformed and divided into smaller pieces, and its degree of swelling decreases over time. According to the nanoliposome-encapsulated SF, the strength of its structure increased and swelling and water absorption decreased. However, a comparison in the swelling ratio of hydrogels containing nanoliposomal SF with the blank nanoliposome showed that the swelling ratio of the blank liposome is lower. Despite the fact that SF is a lipophilic substance, the polar functional groups N=C=S and S=O allow it to absorb water over time, resulting in an increase in its water absorption capacity.

##### pH-Dependent Swelling Behavior of Hydrogel Scaffolds 

**Figure 6:**
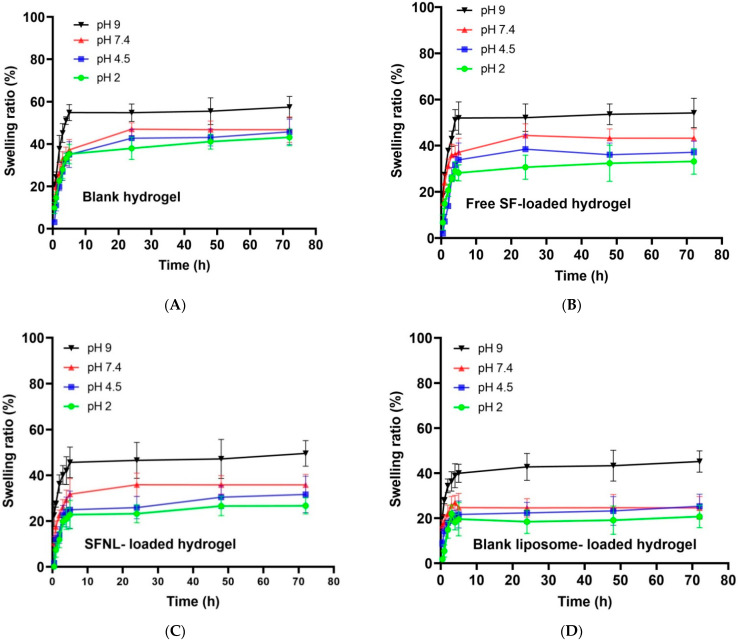
The swelling ratio of freeze-dried PVA/PEG400 hydrogels at different pH; (**A**) blank hydrogel, (**B**) free SF-loaded hydrogel, (**C**) SFNL-loaded hydrogel and (**D**) blank liposome-loaded hydrogel.

The effect of pH on the swelling rate of four samples, namely PVA/PEG400 hydrogels (blank hydrogel, free SF, liposomal SF, and blank nanoliposome encapsulated in the hydrogel was examined in acidic (4.5), neutral (7.4), and basic (9) pH values (Figure 6). It was concluded that all hydrogel samples had the lowest swelling ratio in an acidic environment with pH 2. There was no significant difference when the PVA/PEG400 hydrogel was left at pH 4.5 and 7.4. The maximum swelling ratio at a pH value of 9 (alkaline conditions) reaches 1.5 times the dry weight. This pH-sensitive swelling behavior is related to the structure and ionization constant of acid (pK_a_) and base (pK_b_) ionization groups of hydrogels at the specific pH of the swelling milieu [47]. Liu et al. prepared hydrogels composed of cellulose nanofibrils (CNF) and sodium alginate (SA). The findings showed that the swelling ratio increased by 19 times with the change in pH from 1.5 to 11.0. This pH-sensitive swelling behavior was consistent with our results [38]. In another study, Sabzi et al. designed a drug delivery system based on PVA/citric acid (CA)/Ag nanoparticles (NPs). It was shown that the pH-dependent swelling ratio of hydrogels is strongly influenced by the pH environment, and as the pH of the solution increases, the amount of swelling of hydrogels increases [48]. These similar observations were also reported in our study. This response of swelling behavior to pH changes is related to the degree of ionization and the dissociation constant of free hydroxyl groups of PVA. At pH 2, this group of PVA was protonated [49], and as a result, the amount of hydrogen bonds increased and the electrostatic interaction of PVA decreased and the amount of water absorption decreased. As the pH increased, polar functional groups such as hydroxyl from PVA, N=C=S and S=O from SF, phosphate choline groups from SFNL, and blank nanoliposome changed the state of their ionization, resulting in a decrease in the number of hydrogen bonds and an increase in electrostatic repulsion, which led to a significant increase in the swelling ratio of hydrogels at pH 9 compared to pH 2. On the other hand, it was expected that the swelling of the nanoliposomal form (Figure 6C,D) would increase due to having ionized groups of phosphate and choline at pH 9, but the hydrophobic properties of nanoliposome and the highly created crosslinking which remains rigid that prevented the influx of buffer into the network and swell significantly compared to the free form of SF and blank hydrogel groups (Figure 6A,B).

#### 2.1.7. Mechanical Properties of Hydrogel Scaffolds

**Figure 7:**
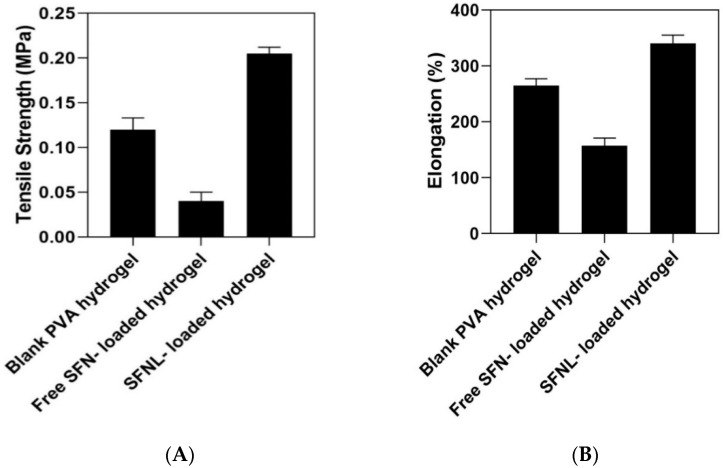
The stress (MPa); tensile strength (MPa) (**A**) and elongation% (**B**) of blank freeze-dried PVA hydrogel, free SF, and SFNL-loaded hydrogel.

Figure 7 displays the mechanical tensile strength of hydrogel scaffolds. This mechanical analysis shows that the tensile strength and elongation of hydrogels are increased by the encapsulation of SFNL. This could be due to the increased cross-linking density in the SFNL-loaded samples. However, tensile strength and elongation at break were decreased in free SF-loaded hydrogel compared to blank hydrogel (*p* > 0.05). In fact, the SFNL-containing hydrogel has a rigid structure with intersections and cross-linking, which excludes it from gel-like behavior. These tensile strength properties of the hydrogel scaffold are necessary to maintain the wound structure and the formation of skin cells [50,51]. 

### 2.2. In Vitro Cell Proliferation

**Figure 8:**
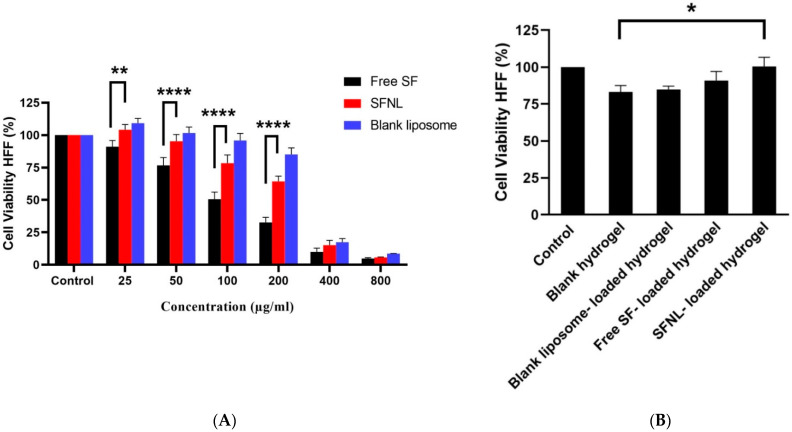
The cell survival assay; (**A**) the cytotoxicity of the free SF, SF-loaded nanoliposomes, and blank nanoliposomes; (**B**) the comparison of the toxicity between the blank nanoliposome-loaded hydrogel, free SF, and SFNL-loaded hydrogel against HFF cells after 48 h. Data were expressed as mean ± SD. * *p* < 0.05, ** *p* < 0.01, **** *p* < 0.0001.

The toxicity of empty nanoliposomes and blank PVA hydrogel on HFF cells was investigated using the MTT technique. Our results indicated that nanoliposome solutions containing less than 200 g/mL of SF and PVA scaffolds containing up to 100 mg/mL were safe (Figure 8). In 2D and 3D scaffold cultures, free SF and SFNL demonstrated concentration-dependent cytotoxicity. The free SF had an IC50 value of 107.2 g/mL, but the SFNL had an IC50 value of 228.8 g/mL, a 2-fold increase over the free form. In other words, SFNL showed less cytotoxicity against HFF cells compared to free SF. At high concentrations, free SF exhibited more toxicity, but at low concentrations, not only was it non-toxic, but it also exhibited antioxidant and tissue regeneration and repair characteristics. Angeloni et al. showed the protective effect of SF and the improved viability of hydrogen peroxide-exposed cardiomyocytes treated with SF (H_2_O_2_) [52]. Tan et al. evaluated the chemopreventive function of SF that stimulate glutathione S-transferase P1 (GSTP1) and NAD(P)H: quinone oxidoreductase 1 (NQO1) as phase II antioxidant enzymes at the mRNA and protein levels in primary normal human bronchial epithelial cells (NHBE). SF enhanced NQO1 protein expression in NHBE cells by up to 11.8 folds, resulting in its protective and antioxidant effects on normal cells [53]. According to the findings of the cytotoxicity assay, the viability of the free SF and SFNL-loaded hydrogel sample was much greater than that of the free SF and SFNL samples. Leng et al. [54] indicated that curcumin-loaded poly(e-caprolactone)-poly (ethylene glycol)-poly(e-caprolactone) (PCEC) nanoparticles diminished the toxicity of curcumin and curcumin/PVA/collagen composite films. According to previous research, they may mitigate the negative effects of curcumin and enhance wound healing [54,55,56]. Of note, the free SF-loaded PVA/PEG400 scaffold may have reversed the toxic effects of the free SF. These findings demonstrated that PVA/PEG400 hydrogel is biocompatible for skin fibroblast cells, increases the adequate and effective dosage of SF by continuous release, and may be employed to expedite the wound healing process. The above results of cytotoxicity correlate well with the cellular uptake findings (Figure 7).

### 2.3. Cellular Uptake 

**Figure 9:**
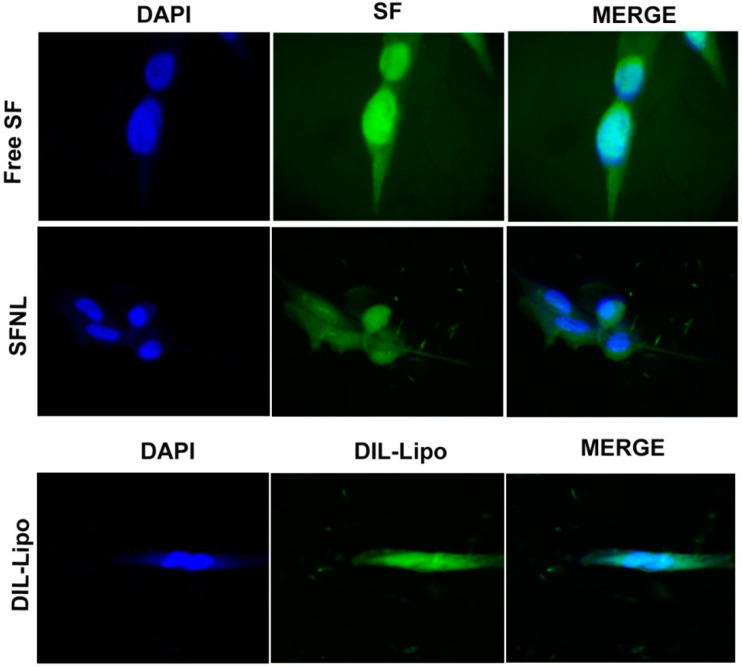
The cellular uptake of free SF, SFNL, and blank liposomes by the HFF cell line over the course of 180 min (60× magnification).

As depicted in Figure 9, the empty nanoliposomes were internalized by the HFF cell line, whereas the amount of free SF accumulated in the cells was less than that of the SF-loaded nanoliposomes. These findings were consistent with cell viability where SF-loaded nanoliposome showed minimal cytotoxicity compared to normal cells. Low cytotoxicity against fibroblast cells is likely due to the slow and gradual release of SF from nanoliposomes inside the normal cell line as compared to the faster release of free SF [57].

## 3. Conclusions

In this study, PVA/PEG400 hydrogel containing SF nanoliposome was developed as a dermal dressing to promote wound healing. The findings indicated that nanoliposomes showed excellent physicochemical properties of particle size with high drug loading and a proper release rate. In addition, PVA/PEG400 hydrogel is able to encapsulate nanoliposomes while maintaining the drug’s properties and long-term protection without disturbing its structure. This dual delivery system represents a sustained drug release method in a pH-sensitive manner. Having knowledge of this response to the pH of the wound milieu enabled us to keep an effective and optimal drug dose with a suitable duration of wound dressing usage without side effects. This hydrogel matrix prevents the wound from drying out and expedites cell-induced tissue regeneration. As revealed from the results, this combined delivery system with sustained drug-release effect could be used as an effective treatment for skin wounds. 

## 4. Materials and Methods

### 4.1. Materials

The sulforaphane solution (2-isopropyl-5-methylbenzoquinone), cholesterol, DSPE-PEG 400 (distearoyl phosphoethanolamine-polyethylene glycol 400), and PVA (polyvinyl alcohol) were purchased from Sigma-Aldrich (St. Louis, MO, USA). DSPE-PEG 2000 (distearoyl phosphoethanolamine-polyethylene glycol 2000)SPC80 (soybean phospholipids with 75% phosphatidylcholine), and Dipalmitoylphosphatidylcholine (DPPC) were obtained from Lipoid GmbH (Ludwigshafen, Germany). The dialysis bag (MW = 12 kDa), PBS (phosphate-buffered saline) tablets, MTT (3–(4, 5-dime-thylthiazol-2-yl)-2, 5-diphenyl tetrazolium bromide), DMSO (dimethyl sulfoxide), chloroform, isopropanol, methanol, and the paraformaldehyde solution, were procured from Sigma-Aldrich (St. Louis, MO, USA). DIL stain (1, 1′-dioctadecyl-3, 3, 3′, 3′-tetramethylindocarbocyanine perchlorate) and DAPI (40, 6-diamidino2-phenylindole) were supplied from Thermo Fisher Scientific (Waltham, MA). Other reagents used in this investigation were of analytical grade without further purifications.

### 4.2. Experimental Design

First, sulforaphane-loaded nanoliposome (SFNL) was prepared and optimized based on different molar ratios of cholesterol: phospholipid. Then, the EE% and release rate at different pH values were investigated. After that, the PVA/PEG400 gel-forming hydrogel system containing SFNL was developed. Finally, the physiochemical properties and cellular viability of optimal formula of SFNL, hydrogel matrix, and SFNL incorporated within hydrogel were evaluated.

### 4.3. Preparation of Sulforaphane-Loaded Nanoliposome (SFNL)

The thin-lipid film hydration method was used to prepare sulforaphane-loaded nanoliposomes. Different molar ratios of cholesterol: SPC80: DPPC were prepared and dissolved in chloroform (as illustrated in Table 1). In all formulations, 5% DSPE-PEG2000 was added. As a hydrophobic drug, sulforaphane (0.5 mg/mL) was dissolved in methanol and added to the lipid mixture in a round bottom flask. Then, the organic solvent was removed by placing the balloon on a vacuum pump at 50 °C on a rotary evaporator (Heidolph, Germany). Finally, the thin lipid film was hydrated by adding a certain volume of the PBS solution at 60 °C for 1 h to attain the liposomal suspensions. The diameter of prepared nanoliposomes was reduced by sonication over an ice bath for 15 min using a micro-tip probe sonicator (E-Chrom Tech Co. Ltd., Taiwan). Unloaded SF was separated from encapsulated SF by a dialysis bag diffusion method (a cut-off of 12–14 kDa) against PBS for 1 h at 4 °C. Then, SFNL was kept in a refrigerator at 4 °C.

### 4.4. Preparation of PVA/PEG400 Hydrogel Scaffold Containing SFNL

PVA/PEG400 hydrogel was prepared by the freeze–thaw method. First, 10% PVA solution was prepared by dissolving PVA powder into distilled water with gentle magnetic stirring on a hot plate for 3 h in a 95 °C water bath at 300 rpm. Then, the temperature of the solution was reduced to 50 °C, and 1% PEG400 was added to it and left for 30 min. In order to develop a PVA solution containing free SF and SFNL, 100 µg/mL SF and 100 µg/mL SFNL were dissolved in the above-formulated PVA/PEG00 hydrogel at 37 °C. The solution was poured into a Petri dish and put through three cycles of 18 h of freezing at -20 °C and 6 h of thawing at room temperature to create interconnected porous structures, strengthen the crystalline and flexible properties of the hydrogel, and reduce its susceptibility to contamination [58]. Then, the produced hydrogel was placed at 4 °C.

### 4.5. Physicochemical Characterization of Synthesized Nanoparticles and PVA/PEG400 Hydrogels 

#### 4.5.1. Size, Distribution, and Zeta Potential of Liposomes

The diameter, polydispersity, and zeta potential of the blank nanoliposome (without drug), as well as liposome-containing SF, were evaluated by the dynamic light scattering (DLS) technique (Zetasizer Horiba Instruments) at 25 °C and the liposome-containing solution was diluted 100 times with deionized water. 

#### 4.5.2. FT-IR Analysis 

Fourier transform infrared spectrophotometer (Model 8300 Shimadzu Corporation, Tokyo, Japan) was carried out to analyze the chemical interactions between SF and the liposome components. The FTIR spectra of the free SF, blank nanoliposomes, SFNL, free SF-loaded hydrogel, SFNL-loaded hydrogel, and blank hydrogel were analyzed in the wavelength range of 400–4000 cm^−1^.

#### 4.5.3. XRD

Crystalline phases of free SF-loaded hydrogel, SFNL-loaded hydrogel, blank nanoliposome-loaded hydrogel, and blank hydrogel were analyzed by XRD (X-Ray Diffractometer, Panalytical EMPYREAN, UK). The intensity scan was recorded at ambient conditions over scattering angles of 2θ = 5°–80° with a step increment of 0.02°/s.

#### 4.5.4. Entrapment Efficiency% (EE%)

The serial dilution of SF with a defined concentration was generated in order to create the standard calibration curve of SF in an isopropyl solution, and its absorption was then measured using UV–VIS spectrophotometry (model T80+, PG Instruments, United Kingdom) at a wavelength of 246 nm (λ max of SF). Afterward, several dilutions of SFNL were prepared in an isopropyl solution and incubated for 30 min at 4 °C to break nanoliposome membrane permitting SF release before measurement. The entrapment efficiency (EE%) of incubated samples was calculated using the calibration curve (R^2^ > 0.99) and the following formula:

EE%=The amount of SF encapsulated within nanoliposome (mg/ml)/Total amount of SF (mg/ml)×100

#### 4.5.5. Release Profile 

In order to determine the release pattern of SF from various liposomal formulations (Chol: phospholipids at molar ratios of 50:50, 30:70, and 10:90), 1 mL of each sample was placed in a dialysis bag (12KD) and immersed in 10 mL PBS with different pH values (4.5, 7.4 and 9) at 37 °C. At a certain time, 1 mL of PBS around the dialysis bag was removed to evaluate the amount of released SF, and then 1 mL of fresh PBS was replaced. The amount of the released SF at different times was quantified using the UV–VIS spectrophotometry at a wavelength of 246 nm (λ max of SF) using a standard calibration curve. In order to assay the release of SF from hydrogel in both forms of free SF and SFNL-loaded hydrogels, 15 mm of hydrogel was immersed in 10 mL PBS at pH values (4.5, 7.4, and 9) and shaken on a stirrer (75 rpm) at 37 °C for 72 h. At specific time intervals (1, 2, 3, 4, 5, 24, 48, and 72 h), 1 mL of PBS was removed and replaced with the fresh one with the same volume. After that, the amount of SF was assessed using a standard calibration curve. 

#### 4.5.6. Swelling Rate Assay

Pieces of hydrogel samples (with a diameter of 15 mm of free SFN-loaded hydrogel, SFNL-loaded hydrogel, blank liposome-loaded hydrogel, and blank hydrogel) were weighed and immersed in PBS at pH values (2, 4.5, 7.4, and 9) at 37 °C. The samples were weighed at various time points (1, 2, 3, 4, 5, 24, 48, and 72 h). The swelling rate was evaluated according to the following formula:

SW% = (*Wt* − *Wi*/*Wi*) × 100

where *Wt* represents the weight of the hydrogel samples at different time points, while *Wi* represents the measured weight of hydrogel samples at the initial time.

#### 4.5.7. Mechanical Analysis 

The mechanical strength of hydrogel samples was measured using a Stable Micro System Texture analyzer (SDL micro 350, Testometric, Rochdale, UK). Briefly, samples were sectioned into pieces (30 × 10 × 3 mm^3^), and the experiments were conducted at a speed of 5 mm/min at 25 °C. The elongation and the tensile strength were evaluated and expressed as the means and standard deviation (mean ± SD).

### 4.6. Cellular Analysis

#### 4.6.1. Cytocompatibility Assay

In order to assess the impact of the free SF, SFNL, blank nanoliposomes, free SF-loaded hydrogel, SFNL-loaded hydrogel, and blank hydrogel scaffold on cell viability, the MTT assay was performed. The HFF cell line was obtained from Medical Nanotechnology and Tissue Engineering Research Center (Yazd, Iran) and cultured in DMEM supplemented with fetal bovine serum (10%), penicillin–streptomycin (1%) and then incubated at 37 °C in a 5% CO_2_-95% air atmosphere. In order to analyze the cytotoxicity of the free SF, SFNL, and blank nanoliposomes, 10^4^ cells were cultured in a 96-well plate to reach 70% confluence; then, they were treated with different formulations at concentrations of 25, 50, 100, 200, 400, and 800 µg/mL. In a 48-well plate, free SF-loaded hydrogel (100 µg/mL), SFNL-loaded hydrogel (100 µg/mL), and blank hydrogel scaffold were added to examine the cytocompatibility of the hydrogel scaffold. After the freezing and thawing process, the plate was exposed to UV light to be sterilized. Then, 2 × 10^4^ HFF cells were cultured on mentioned hydrogels. After 48 h of drug incubation, the MTT solution (10% diluted with DMEM) was added to both 96- and 48 well-plates and left for 3 h. The MTT solution was removed, and 150 µL of dimethyl sulfoxide (DMSO) was added to each well. The absorbance was determined using a microplate reader (Synergy HTX, Bio-Tek, Winooski, VT, USA) at a wavelength of 570 nm.

#### 4.6.2. Qualitative Evaluation of Cellular Uptake

The cellular uptake of the free SF, SF-loaded nanoliposomes, and the blank nanoliposomes labeled with DIL stain was determined using the HFF cell line. Cells were cultured at a density of 10^5^ cells/well in a 6-well plate (Corning, NY, USA) for 24 h. Next, the cells were incubated with mentioned formulations at a concentration of 100 µg/mL for 3 h at 37 °C. Then, the supernatant was withdrawn, and the cells were washed twice with cold PBS and, finally, fixed in 4% paraformaldehyde. The nuclei were stained with DAPI and imaged under a fluorescent microscope (Olympus, Japan). 

## Figures and Tables

**Table 1:** The effect of various molar ratios of cholesterol: phospholipids on EE, as well as the long- and short-term release of the loaded drug.

Formula	Cholesterol: SPC: DPPC: DSPE-PEG2000 (Molar Ratio)	EE%	%Release (6 h)	%Release (24 h)	%Release (48 h)	%Release (72 h)
F1	50:25:25:5	34 ± 7.3	49.6 ± 6.8	53.9 ± 3.9	64.1 ± 5.3	72.3 ± 5.9
F2	30:35:35:5	60 ± 8.4	43.6 ± 5.1	49.9 ± 6.1	61.0 ± 5.4	65.7 ± 6.7
F3	10:45:45:5	88 ± 6.9	31.8 ± 5.4	42.3 ± 5.6	50.3 ± 5.1	57.8 ± 6.0

## Data Availability

Not applicable.
